# Eco-friendly synthesis of carob- and ginger-modified gold nanoparticles for enhanced catalytic applications

**DOI:** 10.55730/1300-0527.3754

**Published:** 2025-07-29

**Authors:** Muradiye ŞAHİN, İlkay Hilal GÜBBÜK, Mustafa ERSÖZ

**Affiliations:** 1 Department of Chemistry, Kırşehir Ahi Evran University, Kırşehir, Turkiye; 2 Department of Chemistry, Faculty of Science, Selçuk University, Konya, Turkiye

**Keywords:** Gold nanoparticles, catalytic activity, green synthesis, heterogeneous catalyst, dyes

## Abstract

This study aimed to investigate the catalytic performance of gold nanoparticles (AuNPs) obtained by using ginger and carob antioxidant plants as reducing and stabilizing agents. In the first stage of the study, AuNPs were successfully produced using these herbal extracts via a green synthesis method, without the need for toxic chemicals, in a sustainable, economical, rapid, energy-efficient, and easy way. In the second stage, the structural analysis and catalytic applications of the obtained AuNPs were investigated. Advanced techniques such as Fourier transform infrared spectroscopy (FTIR), scanning electron microscopy (SEM), transmission electron microscopy (TEM), X-ray diffraction (XRD), UV-Vis spectroscopy, and energy dispersive X-ray spectroscopy (EDX) were used to elucidate the morphological, optical, and structural properties of AuNPs in detail. In UV-Vis analyses, the observation of characteristic surface plasmon resonance (SPR) peaks in the range of 532–540 nm confirmed that AuNPs were successfully synthesized. In the catalytic application dimension of the study, the efficiency of the obtained AuNPs in the degradation of common industrial dyes such as rhodamine B, methyl orange, and methylene blue with NaBH_4_ reduction was investigated. The nanoparticles exhibited high catalytic performance in the reduction of the dyes in question, and the reaction processes were completed in a short time (approximately 8–12 min). The kinetics of these reduction reactions were evaluated within the framework of the Michaelis-Menten kinetic model; important parameters such as reaction rate constants (k) and half-life (t_1/2_) were calculated. The calculated values of K_M_ = 3.45 mg L^−1^ and *v*_max_ = 0.78 mg L^−1^ min^−1^ for Gng-AuNPs reveal that this catalyst exhibits higher affinity for dyes and operates more efficiently at lower concentrations. The findings obtained reveal that AuNPs synthesized using plant antioxidants not only offer an environmentally compatible production process, but also, due to their high catalytic efficiency, provide a potential alternative for the removal of environmental pollutants. In this respect, these nanoparticles offer innovative and applicable solutions in the field of sustainable chemistry and environmental technologies.

## 1. Introduction

Today, metal nanoparticles (MPs) can be successfully synthesized through chemical and physical methods, which are traditional production methods, but these methods include at least one of the important disadvantages such as high cost, toxic chemical content, or high energy and resource consumption [1–4]. In recent years, the green synthesis of nanoparticles (NPs) using biological materials has emerged as a successful alternative to traditional methods [5]. Studies have shown that plants used in eco-friendly synthesis contain flavonoids, saponins, tannins, starch, terpenoids, phenolics, polypeptides, and other biomolecules that play a role in the formation of metal nanoparticles by acting as both reducing and stabilizing agents [6–8]. For example, titanium dioxide (TiO_2_) nanoparticles were synthesized using *Aloe vera* extract and were shown to be effective in the degradation of model paint pollutants [9]. NPs have a wide range of applications, including catalysis, electronics, medical diagnosis, and infection prevention [5,10–12]. The high surface-to-volume ratio of metal nanoparticles, along with their optical, electrical, and chemical-magnetic properties, has dramatically increased their catalytic performance [13,14]. Noble metal nanoparticles with various morphologies have been vigorously investigated for a series of chemical reactions due to their high catalytic activity [15,16]. Gold (Au) is a noble metal, and it has long been a significant material in active research areas such as catalysis, biomedicine, electrochemistry, and spectroscopy [17–20]. Gold nanoparticles, for example, have been used in biosensors and cancer treatment. Therefore, green synthesis of gold nanoparticles using biological materials has great potential for both environmental sustainability and medical applications.

Dye is a colored substance that can be obtained in three different ways as natural, semisynthetic, or fully synthetic [21]. A significant amount of colored wastewater is produced as a result of extensive paint applications in different industries. For instance, textile industry uses large amounts of water during dyeing and finishing processes and produces high volumes of colored wastewater as a result of these processes. For example, approximately 95 to 400 L of water is used to produce 1 kg of textile product. These wastewaters contain high levels of suspended solids (SS), chemical oxygen demand (COD), and toxic pollutants [22]. This wastewater contaminates both groundwater and surface water, posing significant risks to the environment, human health, and aquatic ecosystems [23]. Dyes have a particularly adverse impact on aquatic ecosystems, as they contribute to eutrophication, reduce the oxygen content of water, and hinder sunlight penetration. Moreover, many of these dyes exhibit carcinogenic and mutagenic properties [24–26]. Consequently, there is a pressing need to develop environmentally friendly and cost-effective methods for removing these dyes from water resources and the environment [27].

In recent years, various studies have been carried out on the treatment and recovery of dyes from wastewater in order to contribute to both the protection of water resources and the reduction of water consumption costs of businesses. This study aims to synthesize gold-based nanocatalysts using plant extracts to enhance the degradation of dyes in aqueous solutions, contributing to sustainable water management and environmental protection. Antioxidants extracted from plants impart antibacterial properties to the nanoparticles and prevent their oxidation. As a result, the synthesized AuNPs not only inhibit bacterial growth in water but also effectively remove dyes from industrial wastewater. In this research, three dyes with distinct chemical structures were selected to compare and evaluate the catalytic performance of the synthesized materials: methyl orange (an azo dye, MO), rhodamine B (a xanthene dye, RB), and methylene blue (a thiazine dye, MB). The degradation of these dyes reached 100% within 8–12 min, and they retained their catalytic activity after three cycles. Overall, this study demonstrates that green-synthesized AuNPs exhibit excellent catalytic activity and high efficiency in the comprehensive reduction of organic dye compounds.

## 2. Materials and methods

### 2.1. Materials

HAuCl_4_·3H_2_O (Gold (III) chloride trihydrate) and MB were received from Alfa Aesar (Karlsruhe, Germany). NaBH_4_ (sodium borohydride) and RB were obtained from Sigma–Aldrich (St. Louis, MO, USA), while MO was procured from Merck (New Jersey, USA). Ginger and carob powders were purchased from a local supplier. Double-distilled water (18.2 MΩ) was used throughout the experiments. All materials were of high purity and were used as received without further purification.

### 2.2. Characterization

The composite of ginger and carob and Au nanoparticles (Gng-AuNPs and Crb-AuNPs) was synthesized and characterized in this study. The optical properties of each of the synthesized gold nanoparticles (AuNPs) and their effectiveness in the degradation processes of organic dyes were investigated with a Shimadzu (Kyoto, Japan) UV-1800 model UV-Visible spectrophotometer. This analysis was used to determine the characteristic surface plasmon resonance behaviors of the nanoparticles and to monitor the catalytic degradation process. Fourier transform infrared spectroscopy (FTIR) analysis was performed to determine the functional groups on the surface of AuNPs synthesized with plant extracts; the analyses were performed using a PerkinElmer (Waltham, Massachusetts, USA) 100 ATR model device. In this way, it was aimed to determine the phytochemical compounds that contribute to the stabilization of the nanoparticles. For the analysis of the crystal structure, the X-ray diffraction (XRD) technique was used, and measurements were performed with a Bruker (Rheinstetten, Germany) D8 Advance diffractometer in the 10°–80° 2θ scanning range using a CuKα (λ = 1.5406 Å) radiation source. With this analysis, the crystallinity and phase structure of the obtained nanoparticles were evaluated. Scanning electron microscopy (SEM) and transmission electron microscopy (TEM) were used to reveal the morphological properties of the nanoparticles. While SEM imaging was performed with a Carl Zeiss (Oberkochen, Germany) EVO-LS 10 device to examine the surface topography, high-resolution TEM analyses were performed with a JEOL (Tokyo, Japan) JEM-2100 model device for more detailed examination of the internal structure, size distribution, and shape morphology. This holistic characterization process allowed a comprehensive understanding of the physicochemical properties of the synthesized AuNPs.

### 2.3. Synthesis of AuNPs

**Figure 1:**
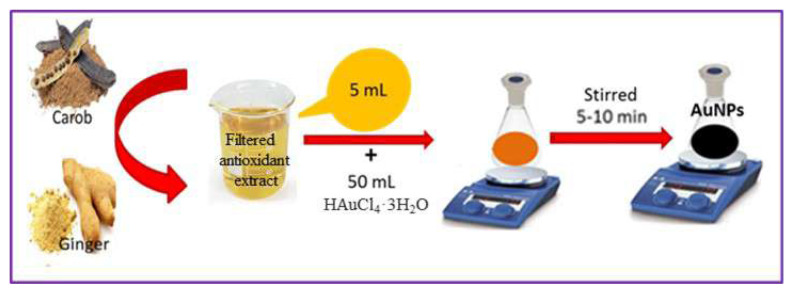
A graphical depiction of the green synthesis of AuNPs using antioxidant extracts.

AuNPs synthesis was carried out in a two-step process under the same optimum conditions (50 mL of 0.1 M the metal salt, 5–10 mL of extract, 298 K, 500 rpm, and 30 min) as described in our previous studies of metallic and bimetallic nanoparticles from carob and ginger antioxidant plant extracts [28,29]. The formation of nanoparticles was confirmed by the distinct color change observed in the solutions (Figure 1), which is an initial indicator of nanoparticle synthesis. This was later verified through detailed UV–Vis spectral analysis. The detailed steps of the AuNPs procedure, which were synthesized at room temperature using antioxidant-reducing agents in a completely environmentally friendly approach, are presented schematically in Figure 1.

### 2.4. Catalytic performance of AuNPs

**Figure 2:**
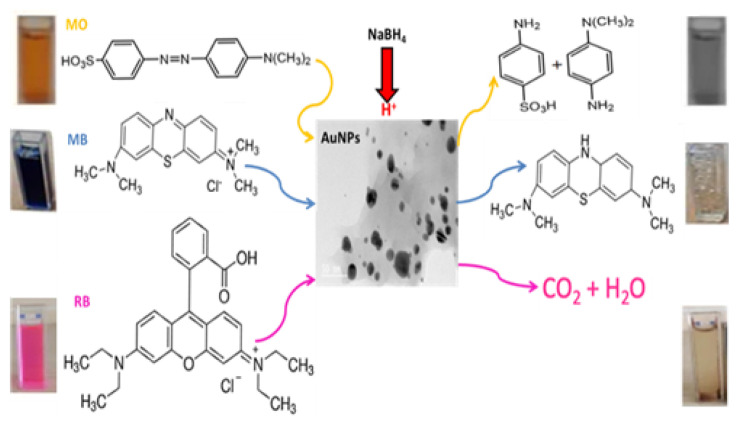
MO, MB, and RB dyes and their degradation products.

The use of NaBH_4_ in the presence of metal nanoparticles is a common method for the reductive degradation of dyes. NaBH_4_ acts as a strong hydrogen source, and electron transfer occurring on the surfaces of metal nanoparticles ensures efficient transfer of this hydrogen to the dye molecules. The catalytic activity of AuNPs was investigated in the presence of NaBH_4_ alone and in combination with NaBH_4_ in the catalytic degradation of MB (664 nm), RB (554 nm), and MO (460 nm) dyes, which have different colors and absorption peaks for their oxidized and reduced structures. The procedure described in our previous study (2 mL of 1 × 10^−5^ mg L^−1^ dye solution, 1 mL of 1 × 10^−2^ mol L^−1^ NaBH_4_, 0.5 mL (2 mg) AuNPs) was applied for the catalysis process [28]. All studies were performed at room temperature in a 4 mL UV cell and concluded with the decolorization of the dyes. Separation of the catalyst from the solution after the reduction is crucial [30], and the water insolubility of AuNPs provides an advantage in that they can be easily separated by filtration. Figure 2 presents detailed spectral changes of the dyes along with their degradation products.

## 3. Results and discussion

### 3.1. Characterization

**Figure 3:**
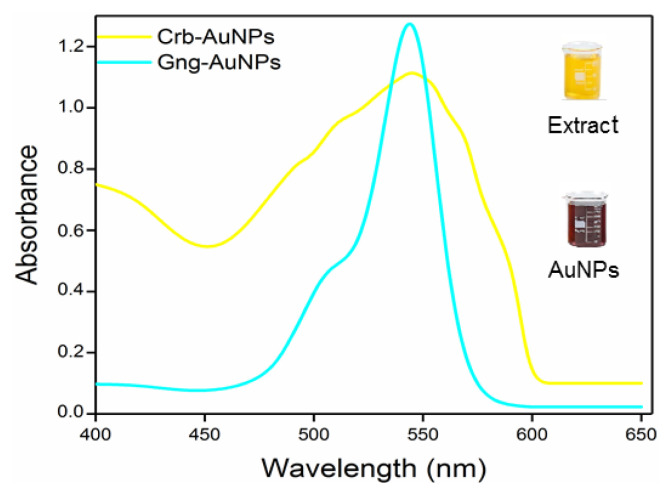
UV-Vis spectra for Gng-AuNPs and Crb-AuNPs.

For comprehensive characterization of the synthesized nanoparticles, FTIR was used to identify functional groups, UV-Vis to study optical properties, XRD and high-resolution transmission electron microscopy (HR-TEM) for morphological and structural analysis, respectively, and scanning electron microscopy coupled with energy-dispersive X-ray spectroscopy (SEM-EDX) to confirm elemental composition. In particular, UV-Vis absorption spectroscopy is one of the fundamental characterization methods widely used to study the optical and structural properties of different metal nanoparticles. The synthesis of AuNPs using antioxidant extracts was identified by observable color changes, where the aqueous solution turned from dark yellow to brown, indicating the formation of nanoparticles. UV-Vis spectral analysis confirmed the presence of AuNPs, as shown in Figure 3, by detecting characteristic absorbance peaks in the 532–540 nm range, which are commonly used to identify gold nanoparticles [3,31,32].

**Figure 4:**
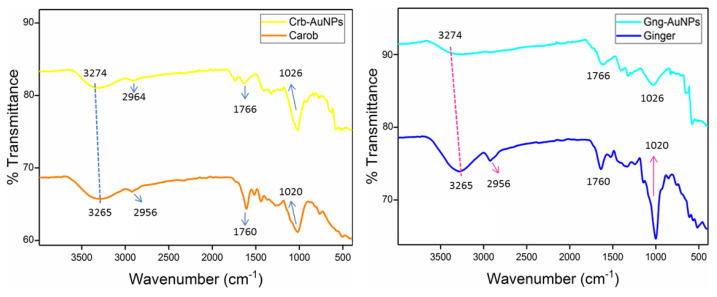
FTIR spectra for plants and AuNPs.

Comparative FTIR spectra of plant extracts and AuNPs synthesized using these plants (Figure 4) revealed that both carob and ginger extracts possess functional groups such as carboxyl, alkalis, methylene, alkenes, and amines. These chemical groups are recognized for their role as reducing agents, facilitating nanoparticle synthesis [8,33]. The FTIR spectra of the plant extracts exhibit characteristic peaks at approximately 1026, 1650, 1766, 2964, and 3274 cm^−1^. The peak at 3274 cm^−1^ corresponds to the stretching vibration of –OH groups, while the peak at 2961 cm^−1^ is attributed to N–H bonds. Additionally, the peaks at 1766 cm^−1^ and 1650 cm^−1^ correspond to C=C aromatic rings or double bonds and C=O groups of carboxylic acids, as well as the stretching of –NH bonds in amide linkages, respectively. Following the formation of AuNPs containing these plants, these peaks exhibit shifts and a reduction in intensity. In the FTIR spectra of each AuNPs, the peaks are observed at approximately 1020, 1760, 2956, and 3265 cm^−1^. These spectral shifts suggest that hydroxyl and carbonyl groups in the plant extracts play a role in the synthesis and stabilization of the nanoparticles [34].

**Figure 5:**
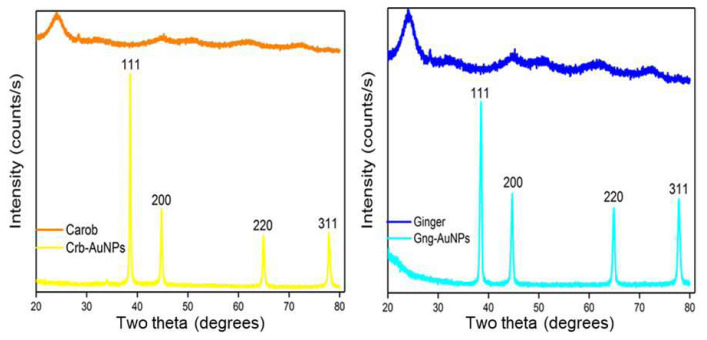
XRD patterns of synthesized AuNPs and plant extracts.

The crystallinity of the synthesized AuNPs was determined by XRD analysis, and its comparison with plant extracts is presented in Figure 5. In the XRD, diffraction patterns revealed peaks corresponding to the (111), (200), (220), and (311) crystal planes, with a prominent peak at 38.2°, 44.8°, 64.8°, and 78.1° angle values for elemental gold (JCPDS 04-0784). From these results, it is understood that the structure is face-centered cubic, indicating the presence of metallic gold [35]. In addition, the absence of additional peaks showed that the prepared gold nanoparticles were of high purity. The crystal size of both AuNPs was calculated using Sherrer’s equation (Eq. 1) based on the strongest (111) plane reflection. The mean crystal size is 25.61 and 23.84 nm for Crb-AuNPs and Gng-AuNPs antioxidant nanoparticle, respectively.


(1) $$D_{111}=\frac{k\lambda }{\left(\beta cos\theta \right)}$$

In this equation, D represents the average crystallite size (nm), λ the X-ray wavelength (0.1541 nm), k the shape factor (k = 0.9 to 1), and θ the Bragg angle (degrees). In addition, β defines the line width at half maximum intensity (FWHM) in radians.

**Figure 6:**
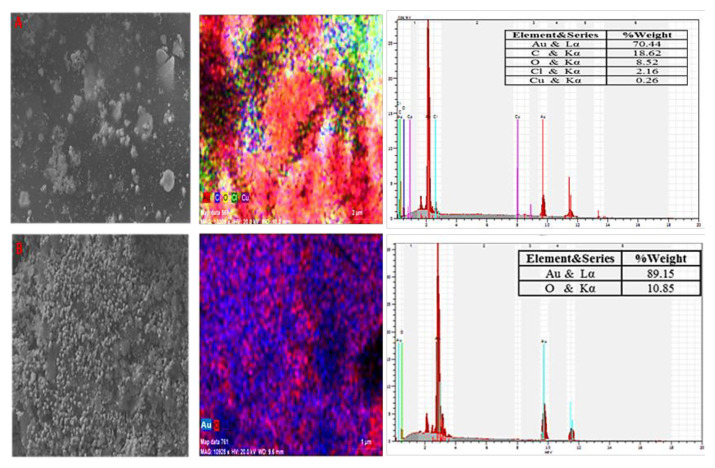
SEM images, elemental mapping, and EDX analysis of (a) Crb-AuNPs, (b) Gng-AuNPs.

Elemental mapping and SEM-EDX analyses were performed to determine the morphological structure and the presence of gold in the nanoparticles. SEM images provided information on surface morphology and particle shape, while EDX provided information on elemental analysis (Figure 6). In addition to Au, elements such as C, O, and Cl were also revealed in the EDX analysis, which is thought to originate from the plant extract used. The presence of copper in the EDX analysis in Figure 6a is thought to be due to the Cu content of the carob. The peaks at 2.10 and 9.70 keV in the EDX spectra indicate gold in nanometallic form. The stronger gold peak at 2.10 keV is due to the matching of the excitation energies of the low-energy electrons with the applied low-acceleration voltage. At the low acceleration voltage, most low-energy electrons and some high-energy electrons are excited [36]. SEM images show that gold nanoparticles generally have an irregular distribution with a spherical shape.

**Figure 7:**
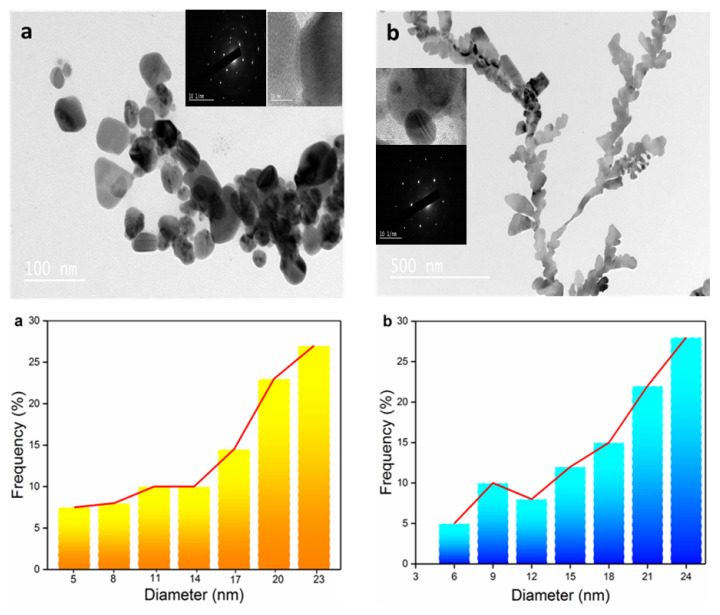
TEM images (HR-TEM analysis and SAED) and size distribution histograms of (a) Crb-AuNPs, (b) Gng-AuNPs.

TEM images clearly demonstrate that the synthesized nanoparticles have a predominantly spherical morphology and occasionally a triangular shape. Figure 7 also presents the size variation histogram of the nanoparticles, revealing an average particle size of 23.26 ± 0.15 nm, with individual particle sizes ranging between 5 and 25 nm. HR-TEM analysis further confirms the presence of well-defined nanoscale lattice features (Figure 7). Furthermore, the selected area electron diffraction (SAED) pattern validates the crystalline structure of the AuNPs. The glowing circular diffraction marks correspond to the (111), (200), (220), and (311) crystallographic planes. The AuNPs exhibit a polycrystalline structure, and as evident from the SAED pattern, they display a single-orientation configuration, resembling a cluster of gold particles. Thus, HR-TEM and SAED analyses confirm that the synthesized AuNPs possess a highly crystalline nature.

### 3.2. Catalytic activity

**Figure 8:**
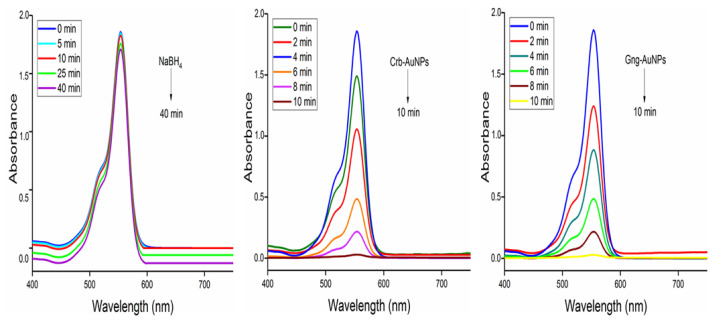
UV-Vis spectra of the NaBH_4_ reduction of RB recorded at 2-min intervals, both without the nanocatalyst and with two types of 0.5 mL AuNPs, respectively.

**Figure 9:**
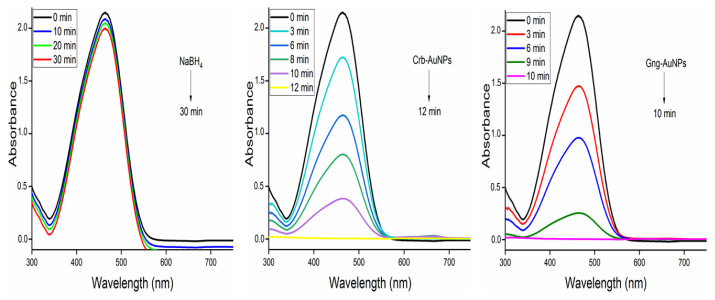
UV-Vis spectra of the NaBH_4_ reduction of MO recorded at 2-min intervals, both without the nanocatalyst and with two types of 0.5 mL AuNPs, respectively.

**Figure 10:**
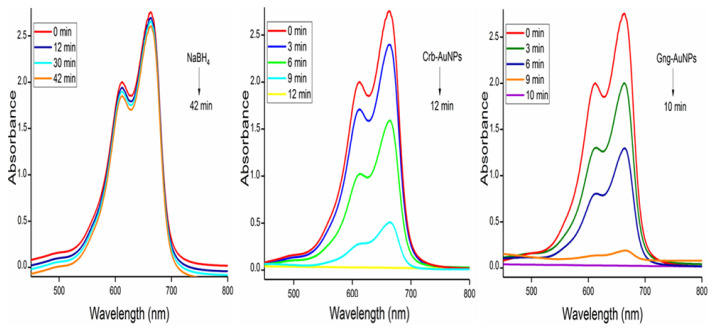
UV-Vis spectra of the NaBH_4_ reduction of MB recorded at 2-min intervals, both without the nanocatalyst and with two types of 0.5 mL AuNPs, respectively.

In the study, two types of AuNPs synthesized with green synthesis approach were used for dye removal with NaBH_4_ via chemical catalysis. Reduction of RB, MO, and MB dyes was carried out at 25 °C using AuNPs synthesized with two different types of antioxidants. The reduction procedure was carried out as explained in Section 2.4. Degradation products of the dyes used are presented in Figure 2. Initially, dye degradation experiments were conducted without adding nanoparticles, namely Crb-AuNPs and Gng-AuNPs. In these experiments, 2 mL of each dye solution was placed in quartz cuvettes, followed by the addition of 1.5 mL of a NaBH_4_ solution (1 × 10^−2^ mol L^−1^). A slight color change was observed in the dyes, indicating partial reduction; however, NaBH_4_ alone was unable to further degrade the dyes even after 1–2 h, as shown in Figures 8–10. In the absence of nanoparticles, NaBH_4_ proved ineffective in fully reducing the dyes. With the addition of Crb-AuNPs and Gng-AuNPs nanoparticles to the medium, complete dye degradation occurred because they facilitated electron transfer between acceptor and donor molecules. The proposed mechanism of electron transfer from NaBH_4_ to the excited MO species, leading to ensuing reduction, is outlined in the succeeding steps [35,37].


$$\begin{array}{l} \text{Au}/{\text{NaBH}}_{4}+\text{h}\surd \to \text{Au}/{\text{NaBH}}_{4}\;({\text{h}}^{+}-{\text{e}}^{-})\\ 2{\text{e}}^{-}+{\text{MOH}}^{+}+{\text{H}}^{+}\to {{\text{MOH}}_{2}}^{-}(\text{Hydrazine derivative}) \end{array}$$

The catalytic performance of Gng-AuNPs demonstrated higher activity in the reduction of all dyes compared to Crb-AuNPs. Although both nanoparticles have similar sizes, the slightly smaller average particle diameter of Gng-AuNPs positively impacted the reaction kinetics. The smaller particle size provided a greater specific surface area, allowing for more effective interaction with reactive molecules. Cu, detected in trace amounts in the EDX analysis of Crb-AuNPs (Figure 6a), may have caused the performance degradation because it can cause atomic alignment defects and electronic interactions that could negatively affect catalytic activity. This difference is considered to be one of the possible reasons why Gng-AuNPs completed the reduction reactions more quickly. The results indicate that natural stabilizers derived from plant extracts play an important role in maintaining the surface morphology and dimensional stability of the nanoparticles. These stabilizers prevent nanoparticle aggregation, ensuring a more uniform distribution and protecting the reactive surface.

**Figure 11:**
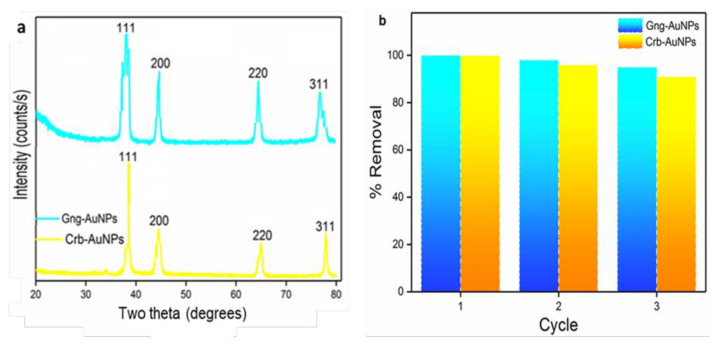
(a) XRD analysis of AuNPs after catalysis and (b) reusability results.

Following catalytic tests, AuNPs were separated from the reaction medium by centrifugation, washed with copious amounts of distilled water to remove surface residue, and then oven-dried at 60 °C. Following three consecutive catalytic cycles to assess their reusability as heterogeneous catalysts, the synthesized AuNPs largely retained both their structural integrity and catalytic activity, as determined by postcatalysis XRD analysis and the removal percentages in each cycle (Figure 11). This demonstrates the potential of bioderived metal nanoparticles for practical applications as environmentally friendly, effective, and reusable catalysts.

### 3.3. Kinetic study

Degradation of RB, MB, and MO was observed to adhere to the pseudofirst-order kinetic model.


(2) $$-\frac{dc_{t}}{dt}=kc,$$

(3) $$\frac{c}{c_{\text{o}}}=Ae^{-kt}+E.$$

**Figure 12:**
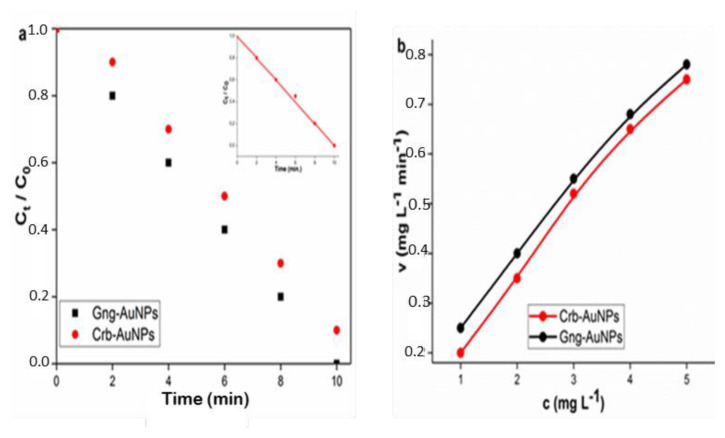
(a) Pseudofirst-order kinetic model for the degradation of RB (b) Michaelis-Menten kinetic curves depicting the degradation of RB by two different types of AuNPs. The presented curves were derived through nonlinear least squares fitting.

**Figure 13:**
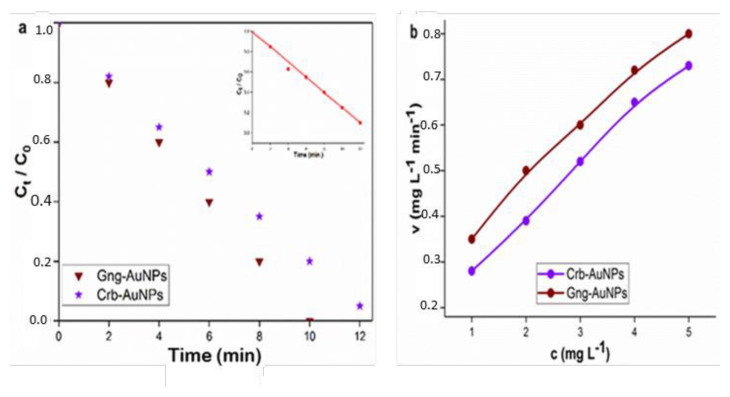
(a) Pseudofirst-order kinetic model for the degradation of MO (b) Michaelis-Menten kinetic curves depicting the degradation of MO by two types of AuNPs.

**Figure 14:**
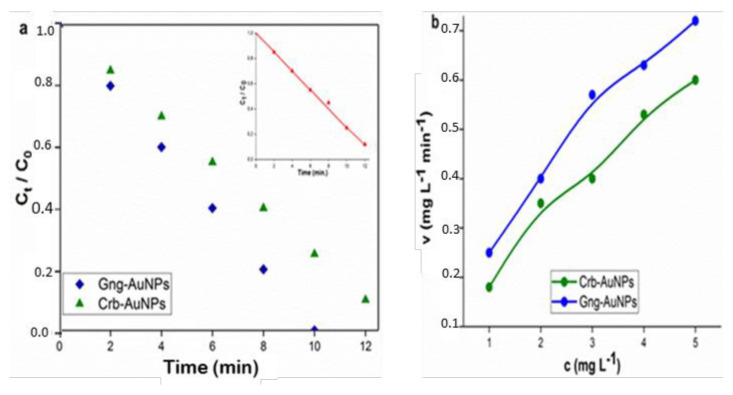
(a)Pseudofirst-order kinetic model for the degradation of MB (b) Michaelis-Menten kinetic curves depicting the degradation of MB by two types of AuNPs.

In this equation, C and C_0_ represent the dye concentrations at times t and 0, respectively. The variable t denotes the reaction time, while k is the reaction rate constant. The standard deviation and rate constant values were determined by the nonlinear least squares method [38], and these processes are shown in Figures 12a, 12b, 13a, 13b, 14a, and 14b and Table 1.


(4) $$t_{1/2}=ln2/k.$$

The half-life of dye reduction (*t_1/2_)* by AuNPs was calculated using Equation 4, and all results are presented in detail in Table 1. Besides, the catalytic reduction reaction was evaluated using Equation 5 based on Michaelis–Menten kinetics [39], and the influence of initial concentration on the reduction rate was determined.


(5) $$\text{V}=\frac{V_{max}C}{K_{M}+C}.$$

Here, c represents the concentration and v denotes the initial velocity, while K_M_ and *v*_max_ correspond to the Michaelis constant and the maximum initial rate, respectively. The starting concentration range for each of the RB, MO, and MB dyes was chosen as 1–5 mg L^−1^, and K_M_ and *v*_max_ values can be derived from nonlinear least squares regression [40]. The dye concentration ranges are illustrated by the Michaelis–Menten curves shown in Figures 12b, 13b, and 14b. From these curves, K_M_ and *v*_max_ were obtained as 6.4520 ± 0.096 mg L^−1^ and 1.5874 ± 0.0096 mg L^−1^ min^−1^, respectively.

Table 2 presents a comparison of the present work with the catalysts reported in the literature. Synthesized AuNPs provided 100% dye removal in a short time and showed almost the same activity after 3 cycles.

## 4. Conclusion

Ginger- and carob-mediated gold nanoparticles (Gng-AuNPs and Crb-AuNPs) were successfully synthesized using a straightforward, low-cost, and environmentally benign green synthesis approach. The successful formation of these nanoparticles was verified through multiple characterization techniques, FTIR, UV-Vis, TEM, SEM-EDX, and XRD. The catalytic efficiency of Crb-AuNPs and Gng-AuNPs was investigated for the degradation of hazardous dyes—RB, MB and MO—in the presence of NaBH_4_ at ambient temperature. The integration of AuNPs with NaBH_4_ significantly enhanced the degradation process, effectively decolorizing all three dyes. The nearly complete decline in absorbance confirmed the high catalytic performance of the synthesized nanoparticles. Both nanoparticle systems exhibited excellent electron transfer capabilities, contributing to accelerated degradation kinetics by reducing the activation energy. Control experiments revealed that NaBH_4_ alone was insufficient to degrade RB, MB, and MO in the absence of AuNPs, underlining the crucial role of the nanocatalysts in facilitating the reduction reactions. Due to the insoluble nature of the AuNPs, they were easily recovered via filtration and subjected to three consecutive catalytic cycles to evaluate their reusability. Postreaction XRD analysis confirmed the retention of the face-centered cubic (FCC) crystalline structure of AuNPs, indicating the preservation of their surface integrity and crystallinity after repeated use. These findings suggest that Gng-AuNPs and Crb-AuNPs, as stable and efficient heterogeneous catalysts, offer strong potential for application in environmentally sustainable remediation technologies.

## Figures and Tables

**Table 1:** Calculated *k* and *R*^2^of catalytic degradation of molecules by catalyst.

Molecules	Catalyst	*k* (min^−1^)	*R* *^2^*	*t* *_1/2_*
RB		0.1367	0.9997	5.07
MO	Gng-AuNPs	0.1201	0.9998	5.77
MB		0.1183	0.9984	5.86

RB		0.1182	0.9898	5.86
MO	Crb-AuNPs	0.1058	0.9912	6.55
MB		0.0994	0.9599	6.97

**Table 2:** Summary of rate constants (k) of different catalysts in the literature.

Molecules	Catalyst	*k* (min^−1^)	*Reference*
MO	AuNPs	0.8	[41]
4NP	Fe_3_O_4_NPs	0.0301	[42]
MB	CuONPs	0.06	[43]
MB	CuONPs	0.0252	[44]
MO	CaONPs	0.0275	[45]
MO	AgNPs	0.3301	[46]
RB	PdNPs	0.0836	[47]
